# Secondary Intention Healing of Extensive Nasal Defects: A Multimodal Approach to Optimize Esthetic Outcomes

**DOI:** 10.1155/crdm/9822266

**Published:** 2026-03-03

**Authors:** Débora Barbosa Rocha Ribas, Yara Tavares Mendonça Garretto, Daniel Gontijo Ramos, Giovanni Indelicato Milano, Gisele Viana de Oliveira

**Affiliations:** ^1^ Post-graduation Department, Faculdade de Ciencias Medicas de Minas Gerais, Belo Horizonte, Minas Gerais, Brazil; ^2^ Residency Program in Dermatology, Surgery Outpatient Clinic, Santa Casa de Belo Horizonte, Minas Gerais, Brazil

**Keywords:** basal cell carcinoma, case report, dressings, laser therapy, wound healing

## Abstract

**Introduction:**

Secondary intention healing (SIH) in the nasal region yields variable cosmetic results depending on the involved subunit. We report a case of a recurrent sclerodermiform basal cell carcinoma (BCC) resulting in an extensive surgical defect involving multiple subunits of the nose, eyelid, and cheek, traditionally requiring complex multistage flap reconstruction, successfully managed with SIH followed by a combination of laser technologies.

**Case presentation:**

A 75‐year‐old male with recurrent sclerodermiform BCC underwent excision resulting in a large facial defect affecting multiple nasal, eyelid, and cheek subunits. The wound was managed with SIH supported by sequential use of advanced dressings. After several weeks, the patient developed a small hypertrophic scar along the lateral nasal sidewall and malar region. A combined protocol using intense pulsed light (IPL), a fractional ablative erbium laser, and 5‐fluorouracil (5‐FU) drug delivery resulted in progressive remodeling and complete clinical resolution of the hypertrophic component, yielding a nearly imperceptible scar.

**Discussion:**

Early postoperative intervention with a multimodal laser approach may significantly enhance cosmetic outcomes following SIH, even in extensive defects involving multiple facial subunits. This case illustrates the potential of combined laser technologies and 5‐FU as a minimally invasive strategy to optimize scarring and reduce the need for complex reconstructive surgery.

## 1. Introduction

Secondary intention healing (SIH) in the nasal area leads to varying cosmetic outcomes depending on the subunit area treated [1]. When dorsal nasal area defects are treated with SIH, 67% of scars result in acceptable outcomes, while unacceptable results include depressed, hypopigmented, or hypertrophic scars (HSs). Large defects involving multiple nasal subunits generally result in poor outcomes [2].

We report the case of a patient with an extensive basal cell carcinoma (BCC) treated in a public healthcare setting, in which excision with controlled margins (Figure 1(a)) was performed due to the unavailability of Mohs micrographic surgery. The resulting surgical defect (Figure 1(b)) was managed by SIH, initially using calcium alginate dressings, appropriate for phases of greater exudation, and subsequently, after 15 days, hydrocolloid dressings. The patient progressed with adequate epithelialization but developed a retractile HS (Figure 1(c)).

FIGURE 1Visual documentation of basal cell carcinoma (BCC) management. (a): Extensive basal cell carcinoma; (b): basal cell carcinoma excision with controlled margins, managed by secondary intention healing initially with using calcium alginate dressings, appropriate for phases of greater exudation, and subsequently, after 15 days, hydrocolloid dressings; (c): retractile hypertrophic scar; (d): clinical improvement after a combination of intense pulsed light (IPL) and dual‐mode ablative fractional Erbium:YAG 2940 nm laser (Vydence), followed by drug delivery of 5‐FU.

**Figure:**
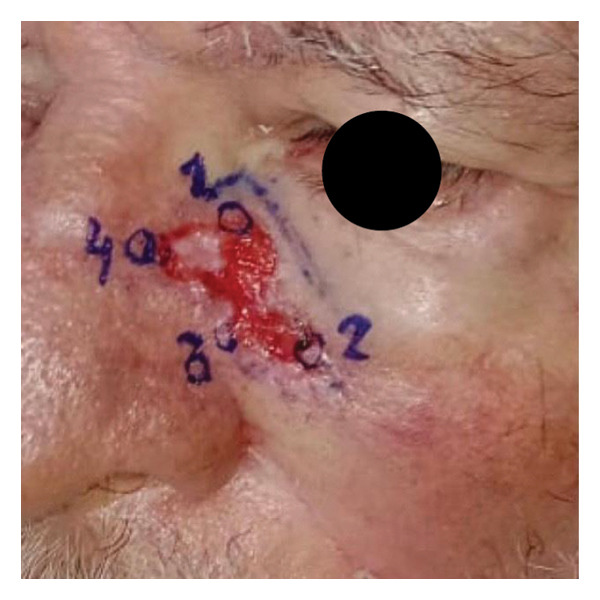
(a)

**Figure:**
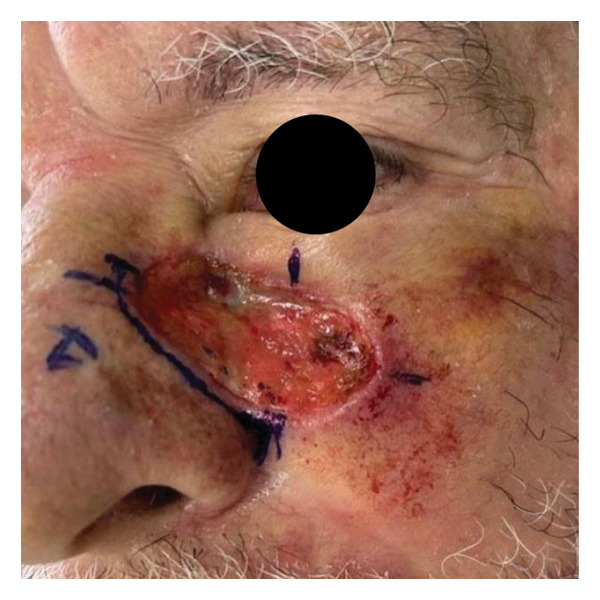
(b)

**Figure:**
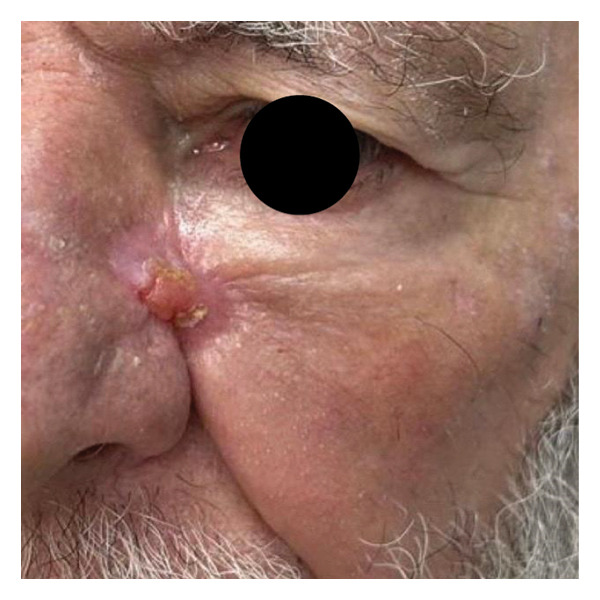
(c)

**Figure:**
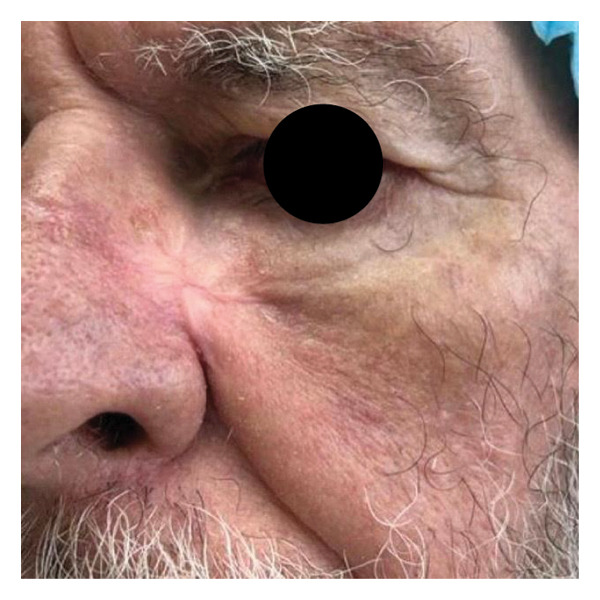
(d)

In response to this HS, we initiated conservative treatment using a combination of intense pulsed light (IPL) and dual‐mode ablative fractional Erbium:YAG 2940 nm laser (Vydence), followed by drug delivery of 5‐FU. After two sessions, the patient exhibited remarkable clinical improvement (Figure 1(d)), and no further surgical intervention was required.

This case illustrates that SIH may represent a valuable and cost‐effective reconstructive option in selected clinical scenarios [3, 4]. Its adjunctive use with lasers refines the healing process, providing acceptable cosmetic outcomes.

Our objective was to document the improvement achieved with these emerging approaches in an anatomically concave region, where traditional surgical reconstruction would likely require complex flaps involving multiple facial subunits.

## 2. Case Presentation

A 75‐year‐old male had a recurrent BCC operated on by our team, resulting in a large defect encompassing multiple subunits of the nose, eyelid, and cheeks.

An SIH approach was chosen to avoid complex and multiple surgical steps. A highly absorptive dressing (calcium alginate sheet) was applied during the first postoperative week, anticipating a secretory wound. The alginate sheet was then replaced with an extra‐thin hydrocolloid dressing, which was changed weekly for 2 months. The patient later returned with a HS on the lateral nose sidewall and cheek area. We then administered two monthly sessions combining a 2940 erbium fractional ablative laser and IPL (Vydence) followed by drug delivery of 5‐FU (Faldfluor 50 mg/mL). Drops of the 5‐FU solution were administered from a 3 mL syringe to the laser‐treated area immediately after the fractional procedure. The parameters used were erbium: Dual Mode, 15/15, 300/3; IPL: filter 540, 14/10 (first session) and 640 wavelength, 15/15 (second session). Hydrocolloid dressings were maintained until the end of the treatment, 5 months after surgery.

The patient was highly adherent to the treatment. This combination of technologies, drug delivery, and hydrocolloid dressings resulted in an outcome that surprised our team with an almost invisible scar.

At the conclusion of the treatment, the patient reported extreme satisfaction with the esthetic outcome. The patient further expressed that neither he nor his family members could identify the area where the tumor was located.

## 3. Discussion

The immediate treatment of a surgical wound managed with SIH involves maintaining a moist environment [5], which can be achieved using antiseptic solutions and various dressings. Among the approaches for treating wounds healed by secondary intention, moist healing environments are recommended. Occlusion promotes granulation tissue formation and reepithelialization and restores epidermal barrier homeostasis, signaling dermal fibroblasts to reduce collagen synthesis [6, 7]. Antiseptic solutions and petrolatum or equivalent ointments are advised. However, using dressings that can remain in place for several days offers an easier recovery for the patient. It has been previously demonstrated that hydrocolloid dressings can treat HSs, yielding results that are comparable to silicone dressings [8].

SIH can be a viable option with satisfactory esthetic results when primary defects present a reconstructive challenge, as recent randomized study demonstrates that it often results in better patient‐reported outcomes regarding color and texture matching compared to skin grafts [9]. In this study, we have shown that a combination approach including dressings and lasers allows for positive cosmetic outcomes. Complications following SIH of multiple subunit areas of the face include ectropion, HSs, and delayed healing [10].

Given the large size of this patient’s defect, we anticipated a significant HS. However, the use of occlusive techniques with hydrocolloid dressings, as described elsewhere [8], contributed to a favorable outcome, with no hypertrophic scarring. At the final follow‐up, clinical examination confirmed the absence of scar contracture or anatomical distortion, such as asymmetry of the nasolabial fold, thus maintaining the patient’s natural facial contours. We believe that the combination approach shown in this study offers a new perspective on SIH. Despite the favorable outcome, this case report has inherent limitations. As a single‐case observation, the generalizability of this specific multimodal protocol is limited, and the 5‐month follow‐up is insufficient to confirm long‐term esthetic stability.

## Funding

This article has no funding source.

## Ethics Statement

The study adhered to the ethical guidelines of the Declaration of Helsinki.

## Consent

The patient in this study has given written consent for the publication of this case report, including the associated images.

## Conflicts of Interest

Gisele Viana de Oliveira reports receiving equipment discount and support for attending meetings and/or travel from Vydence and LMG (laser industries). The other authors declare no conflicts of interest.

## Supporting Information

A. Care checklist for case reports.

## Supporting information


**Supporting Information** Additional supporting information can be found online in the Supporting Information section.

## Data Availability

The data for this study are available upon request.
